# School feeding in Ethiopia: a scoping review

**DOI:** 10.1186/s12889-023-17613-4

**Published:** 2024-01-10

**Authors:** Samson Mideksa, Tsegaye Getachew, Firmaye Bogale, Ermias Woldie, Desalegn Ararso, Aregash Samuel, Meron Girma, Masresha Tessema, Mamuye Hadis

**Affiliations:** 1 Knowledge Translation Directorate, Ethiopian Public Health Institute, P O Box 1242, Addis Ababa, Ethiopia; 2 Food Science and Nutrition Research Directorate, Ethiopian Public Health Institute, Addis Ababa, Ethiopia

**Keywords:** School feeding program, Child undernutrition, Nutritional outcomes, Academic performance, Coverage, Quality, Funding issues, Ethiopia

## Abstract

**Introduction:**

Undernutrition is a major public health problem in developing countries, especially in Sub-Saharan Africa. Undernourished children are smaller and have low weight. To solve this issue, school feeding (corn-soya blend, vegetable oil) started in 1994 in Ethiopia. Thus, this scoping review aims to map the evidence relating to school feeding programs and their potential role in managing children`s nutrition in Ethiopia.

**Methods:**

This scoping review is informed by the methodological framework of Arksey & O’Malley for scoping reviews and recommendations on the framework by Levac and colleagues. The databases searched included the Education Resources Information Centre, International Initiative for Impact Evaluation, Cochrane Library, MEDLINE, and Google Scholar. To ensure its comprehensive search, grey literature sources were searched. The search was undertaken on 26 April 2023. Studies on school feeding, such as coverage, and studies that evaluate the educational and nutritional impacts of school feeding in Ethiopia, regardless of study designs, were included. Reports (publications) about school feeding without scientific methodology were excluded.

**Result:**

Twenty-seven studies were included in this review. It includes cross-sectional, prospective cohort, laboratory-based analysis, experimental, case study, and qualitative study designs. The school feeding program results were inconclusive, while some indicate a positive effect on body mass index, height, thinness, anemia, weight, dropout rate, class attendance, and enrollment. The others showed that the school feeding program did not affect stunting, thinness, weight, hemoglobin level, enrollment, attendance, dropout rate, and academic achievement. Factors affecting school feeding programs negatively include poor quality food and financial constraints. However, no literature on school feeding program coverage was found.

**Conclusion:**

School feeding programs improved nutritional status, and academic performance, although some studies show any effect. Poor-quality food provisions and financial constraints affect school feeding programs. There are mixed findings, and further research is required to determine the effect of school feeding programs conclusively. To ensure the program's sustainability, it should be supported by a national policy, and budget allocation is needed. In addition, more evidence should be generated to show the coverage of school feeding programs in Ethiopia.

## Background

 Undernutrition causes eight million child deaths worldwide every year [1] It is a major public health problem in developing countries, especially in Sub-Saharan Africa [2] Despite some positive progress in economic growth observed in many developing countries, undernutrition continues to be highly prevalent and is associated with poor health status and poor academic performance [3]. Undernutrition inhibits academic attainment through poor growth and mental development, reduced motivation and poor cognitive development [4]. On the other hand, being overweight and obese is reported to have the potential to impair academic performance via social pathways such as discrimination and stigma [5]. According to the Global School-Based Student Health Survey, the mean body mass index (BMI) estimates among adolescents in South Asia, Southeast Asia, East Africa, West Africa and Central Africa are < 20. The lowest age-standardized mean BMIs were seen in Ethiopia, Niger, Senegal, India, Bangladesh, Myanmar, and Cambodia [6]. The World Bank report also indicated that, academic performance in students of Sub-Saharan African countries is less than half of what is expected for their age based on the Africa Student Learning Index (ASLI) [7]. Ethiopia is among the countries where adolescent students’ academic achievement is low according to the ASLI score measured [7, 8]

School age provides an opportunity to remedy nutritional and developmental deficits that were not addressed during early childhood [9]. Nutritional interventions in school-aged children have been reported to result in improved cognitive function [10–12]. The School Feeding Program (SFP) has been recognized as a platform for nutritional, health and educational intervention programs [13]. The contribution of SFP with regard to outcomes of energy intake, micronutrient status, enrollment and school attendance and academic achievement displayed relatively consistent positive effects [14, 15]. Its positive effect on physical growth, cognitive and academic performance was less conclusive in some countries while substantial effect was seen elsewhere [16, 17]. The SFP is also believed to pave the way to achieve sustainable development goals and to reduce inequalities in education. In sub-Saharan African countries, SFP showed an encouraging effect on learning outcomes and a small average effect on attendance [18]. In Ethiopia, evidence on the nutritional and educational effects of SFP is minimal with some pocket studies conducted at the sub-national level [19] The Ethiopian school feeding programme, which has been in operation for 30 years, is expanding its reach and putting more strategic emphasis on developing a pilot project that connects school feeding with regional agricultural production. The Ethiopian government is actively working to change the nation's agricultural sector, including its approaches to school feeding, through effective policies and projects [20].

This scoping review aims to map the evidence relating to school feeding programs and their potential role in managing children under nutrition. Thus, its main focuses are to explore coverage of school feeding, quality of school feeding, nutritional impacts, funding issues, and effects on educational achievements among students in Ethiopia. The study results will inform the public, donors, academia, policy makers and other stakeholders. To make judicious use of evidence regarding school feeding, scientific evidence regarding school feeding in the country should be summarized, analyzed and presented in an accessible format.

## Methods

The scoping review used the methodological framework of Arksey & O’Malley for scoping reviews [21]. and recommendations on the framework by Levac and colleagues [22]. This framework has six stages: 1) identifying the research question; 2) identifying relevant studies; 3) study selection; 4) charting the data; 5) collecting, summarizing and reporting the results; and 6) consultation with relevant stakeholders. A protocol was developed and registered on Open Science Framework (OSF) on March 30, 2023 https://osf.io/5m6dh/ as OSF preregistration.

### Identifying relevant studies

The following databases were searched to find relevant studies: Education Resources Information Centre (ERIC), International Initiative for Impact Evaluation (3ie), Cochrane Library, and MEDLINE. In addition, Google Scholar was used. Furthermore, to make the search as comprehensive as possible, grey literature sources were searched. These sources include databases of relevant organizations such as the World Health Organization (WHO), the World Food Programme (WFP), the Food and Agriculture Organization (FAO), China Foundation for Poverty Alleviation, United Nations International Children's Emergency Fund (UNICEF), United Nations Educational, Scientific and Cultural Organization (UNESCO), Addis Ababa University electronic library, and website of the Ministry of Education. Only studies published or written in English without date limits were considered. The search strategy included all identified keywords and index terms from MESH terms for the included database and/or information source. The reference lists of all included sources of evidence were screened for additional studies. The first search was undertaken on 30 April 2022 and updated on 26 April 2023.

### Study selection

Studies on school feeding program or its impacts among Ethiopian students regardless of study design were included. Studies on both sexes and any form of school feeding intervention in the school compound were included. Surveys related to school feeding were also included. Reports that lacked scientific/systematic information and outcomes in terms of nutritional and educational outcomes were excluded.

The review process had two levels of screening: title and abstract review and full-text review. Articles retrieved were screened independently by two groups of reviewers (SM & EW) and (TG & DA) to assess eligibility, as determined by the inclusion criteria. Full copies of all potentially eligible papers were retrieved. Disagreements at any of the eligibility assessment processes were resolved through discussions and consultation with the team (MH & FB) where necessary.

### Data extraction

Relevant information from selected studies was extracted using a form developed by the team. The data collection form included information on: author(s), year of publication, study design, and key findings as they relate to the scoping review question. Data extraction was independently conducted by two groups of reviewers. Differences among reviewers were resolved by involving the other reviewers from the team.

## Results

### Literature search

**Fig. 1:**
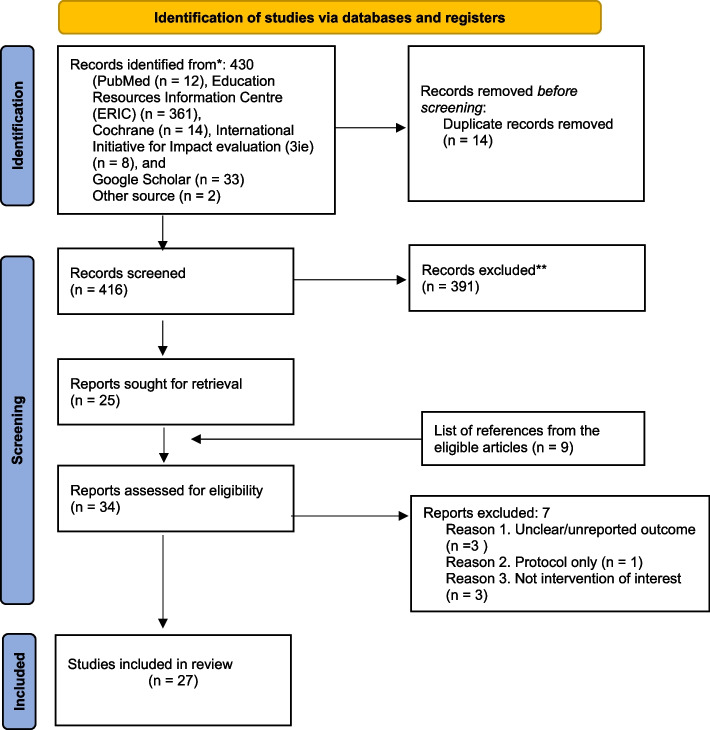
PRISMA flow diagram for the scoping review process

The literature search in this review yielded 430 records from all the databases (Fig. 1). After removing duplicates and screening the titles and abstracts, 405 studies were excluded. Before going into full text screening, nine articles were additionally included from the reference of selected studies. Hence, the full texts of 34 potentially eligible studies were retrieved, and, seven studies were excluded.

### Excluded studies and reasons for exclusion

The studies excluded and their reasons for exclusion are summarized in Table 1 [23–29]. Seven studies were excluded among the selected full text articles. Studies were excluded for not looking at outcome of interest (2), not looking at intervention of interest (4) and a study that was only at a protocol stage (1).

**Table 1:** Excluded studies and reasons for exclusion

No	Authors	Year	Reasons of exclusion
1	Appleby et al. [23]	2019	The study was a review and did not show the clear outcome
2	Keating et al. [24]	2019	The study was on integrating school health and nutrition program perception
3	Kim SS, Menon P, and Tadesse A. [25]	2021	The paper was a protocol
4	Okello et al. [26]	2022	The study was about fortified food
5	Blom SC. [27]	2014	The study did not have relevant outcome for this study
6	Belayneh et al. [28]	2018	The study was not about school feeding program
7	Destaw et al. [29]	2021	The study assessed baseline and did not assess the effect of school feeding

### Characteristics of included studies

All included studies were carried out between 2011 and 2022. The majority of the studies (19) were cross-sectional, while the remaining four were cohort studies, one was an experimental study, one was a case study, and another was a laboratory- based study. This scoping review examined the range and nature of literature on school feeding. The studies on school feeding in the Ethiopian context have covered different aspects including coverage of school feeding, quality of school feeding, nutritional outcome, funding issues, and effect on educational achievements among students in Ethiopia. The majority of the identified studies were focused on the educational impact of SFP.

### Studies on nutritional impact

Ten of the studies included in the review had the nutritional status of SFP. These studies included cross sectional, cohort and lab-based analysis studies. The studies showed mixed results. Two cross sectional studies showed that SFP increased BMI, [30, 31] another two studies found that SFP decreased thinness, [32, 33]. two studies found that children who consumed school meals had reduced anemia, [34, 35] one study found that SFP reduced underweight, [33] and another study found that height was increased among children in SFP [30]. In contrast, a study reported that the prevalence of stunting was greater among students who ate lunch at school, even though this difference was not statistically significant once the relevant confounders were taken into account [32]. Similarly, a study reported that caloric and nutritional contributions were less than two-thirds of the daily reference nutrient intakes (RNIs) needed from school meals, except those of fiber, thiamine, calcium (for early adolescents), and iron [36]. Other studies found no effect on stunting, [35, 37] thinness, [37] anemia, [38] weight, [33] and height [35]. Generally, seven of the studies reported favorable results on the importance of SFP for nutritional status [30–36] Table 2 summarizes the main aspects of the studies.

**Table 2:** Summary of studies on the nutritional impact of school feeding programs in Ethiopia

No	Authors	Year	Aims/objective of the study	Study design	Key findings on Nutritional impact
1	Zenebe M, Gebremedhin S, Henry C J, and Regassa N. [30]	2018	To examine effects of SFP on dietary diversity, nutritional status and class attendance of school children	A school-based comparative cross-sectional study	SFP beneficiaries had higher mean values for dietary diversity score, body mass index for age (BAZ), and height for age (HAZ)
2	Ayehu SM and Sahile AT. [31]	2021	To assess the Body Mass Index and factors associated with School Absenteeism	Cross sectional	School feeding significantly increases average weight and BMI
3	Demilew YM, and Nigussie AA. [32]	2020	To compare the nutritional status of primary school students enrolled in schools with school feeding programs and in schools without school feeding programs and to identify associated factors	Cross-sectional study	School feeding significantly reduces thinness. However, higher prevalence of stunting was greater among students who ate lunch at school, albeit it was not significant after adjusting for the potential confounders Students who did not take meal at school were positively associated with thinness
4	Desalegn TA, Gebremedhi S, and Stoecker BJ. [38]	2022	To assess the effect of SFP on the anthropometric and haemoglobin status of school children	Prospective cohort study	School feeding does not have effects on weight, height and hemoglobin concentration
5	Destaw Z, et al,. [36]	2022	To investigate the nutritional quality and adequacy of school meals served to school-age children and adolescents	Lab based analysis	The caloric and nutritional contributions were generally less than two-thirds of the daily RNIsneeded from school meals, except that of fiber, thiamine, and calcium (for early adolescents), and iron
6	Mekuria, D.D., Alemu, Z.A. and Abaerei, A.A. [33]	2021	To explore the association between school meals program and a child’s nutritional status	A comparative cross-sectional study	Children attending schools without the school feeding program had greater rate of underweight and wasting
7	Lakew B. [39]	2021	To investigate the adequacy of meal macronutrients, dietary diversity, body composition, and the iron status of school children aged between 7–14	A cross-sectional study	The overall body composition of the schoolchildren was not related to the meals served by the school food program Food variety and dietary diversity scores among schoolchildren fell short of the Food and Agriculture Organization's suggested levels
8	Mohammed H. [37]	2018	To compare nutritional status and associated factors in the selected primary schools children with or without school feeding program	A school based comparative cross sectional	There were no statistically significant differences in stunting and thinness among beneficiary and non beneficiary children
9	Mokonnen G. [34]	2017	To assess the impact of school feeding program on nutritional status and academic performance of children	A School based cross sectional and comparative perspective cohort study	School fed intervention reduced anemia
10	Tadesse K. [35]	2018	To evaluate the effect of school feeding programs on anemia and stunting levels among school feeding beneficiaries and on evaluating meals for adequacy in nutrient contribution	A Quasi Experimental Study	Anemia was lower in students in the school feeding program No difference on stunting Iron contribution from school meals was adequate for all age and sex groups except for girls in the age group of 10–14 years

### Studies on educational impact

Nineteen studies reported on educational impact (Table 3). The studies showed mixed results. Some of the findings conclude that the implementation of a school feeding program enhances academic achievement, [35, 40–42] raises class attendance, [30, 31, 40, 43–50] reduces dropout rates, [42–44, 49] and increases enrollment. [40, 42, 48, 50] On the other hand, a study reported that stopping SFP increases male enrollment, and decreases class repetition [49]. In contrast, there were also studies that reported that, there is no significant effect of SFP on the dropout rate, [34, 48, 51, 52] academic achievement, [46, 53, 54] and attendance [34, 48, 51, 53, 54]. The majority of studies (12 studies) have reported favorable results on the importance of school feeding programs for educational outcomes.

**Table 3:** Summary of studies on the educational impact of school feeding programs in Ethiopia

No	Authors	Year	Aims/objective of the study	Study design	Key findings on Educational impact
1	Ayehu SM and Sahile AT. [31]	2021	To assess the Body Mass Index and factors associated with School Absenteeism	Cross sectional	School feeding significantly reduces school absenteeism
2	Delbiso TD, Kotecho MG, Asfaw FM, and Fekadu Mulugeta. [43]	2021	To explore the effects of COVID-19 imposed interruption of the Addis Ababa school feeding program on students	Qualitative research	The school feeding program increases school attendance and in-class concentration and minimizes lateness and dropout rates
3	Desalegn TA, Gebremedhin S, Alemayehu FR, and Stoecker BJ. [44]	2021	To evaluate the effect of the SFP on class absenteeism and academic performance of primary school students (grade 5–8)	Prospective cohort study	Students not in SFP are twice as likely to miss classes and six times more likely to drop out of school
4	Desalegn TA, Gebremedhin S, and Stoecker BJ. [45]	2022	To explore the successes and challenges of the SFP	Exploratory qualitative study	Homegrown school feeding improves class attendance and academic performance
5	Destaw Z et al,. [40]	2022	To evaluate the impact of the Addis Ababa School Feeding Program (SFP) on educational outcomes	Single-group repeated measurement/longitudinal study design	SFP improved educational outcomes, teaching–learning environment, student enrollment, and attendance
6	Robert P, Markus F and Getinet H. [41]	2017	To investigate the relationship between providing school meals programmes and educational out-comes	Descriptive study	On-site school meals supplemented with take-home rations improve educational outcomes. Morning meals help students than those provided at the end of school
7	Assefa M. [42]	2022	To investigate the challenges and impacts of primary school feeding	Descriptive survey research design	School feeding program promotes enrollment and lowers drop-out rates
8	Dheressa D K. [51]	2011	To investigate the impact of school feeding on school enrollment, class attendance, and student drop-out patterns among primary school children	A case study	The School Feeding Program did not significantly improve enrollment, attendance, or dropout rates
9	Aregawi F. [52]	2012	To evaluate the impact of school feeding program on student enrollment and dropout and describe constraints that hamper its effective implementation	Descriptive quantitative and qualitative	Student dropout rate was unaffected significantly by the school meal program
10	Seyoum IA. [53]	2021	To assess the effect of School Feeding School Input Supply Program on the school performance of primary public-school children	Quantitative evaluative and qualitative research approaches	The effects of the school feeding program on academic attainment and attendance rates were insignificant
11	Assefa E. [46]	2015	To investigate the impact of school feeding program on school performance specifically on students’ achievement Test score and students’ class attendance rates among primary school children	Experimental research design	There was no significant difference in achievement test scores There was significant difference in attendance rate
12	Gebreamlak B. [47]	2018	To assess the impact of school feeding program on school participation and dietary intake of children	Descriptive research design	Significant increase in attendance, improvement in learning interest, punctuality, participation in tutorials, advancement in participation in extracurricular and curricular activities
13	Guta A. [48]	2014	To assess effect of school feeding program on school enrollment, class attendance and drop-out	Community based comparative cross-sectional study	SFP improved enrollment, but no significant improvement on school attendance, and dropout rates
14	Mokonnen G. [34]	2017	To assess the impact of school feeding program on nutritional status and academic performance of children	A School based cross sectional and comparative perspective cohort study	High academic performance among the school feeding group No significant difference in rate of absenteeism, dropout and class repetition
15	Gallenbacher D I H. R. [49]	2018	To evaluate the effect of stopping a school feeding programme on access to education (enrollment, drop-out and attendance rates) and learning achievement (repeater rates)	A School based cross sectional and comparative perspective cohort study	School feeding phase increased dropout of girls, reduced attendance rate, and reduce class repetition. However, it significantly increases in male enrollment
16	Haile Y. [50]	2019	To explore the sustainability of school feeding program	Descriptive quantitative and qualitative	SFP raises student morale and educational aspirations, enhances enrollment and attendance, pastoral parents' understanding of and appreciation for education, and raises educational standards
17	Yohannes A. [54]	2017	To assess the effect of School Feeding Program on the school performance of primary public school children	A quantitative evaluative research approach and a quasi-experimental design	No significant impact of school feeding on academic achievement, attendance and children’s attention
18	Zenebe M, Gebremedhin S, Henry C.J, and Regassa N. [30]	2018	To examine the effects of SFP on dietary diversity, nutritional status and class attendance of school children	A school-based comparative cross-sectional study	Non beneficiaries of SFP missed substantially more school days on average

### School feeding on other variables

Few studies have reported on the quality of food [39, 45, 55] in SFP programs and financial or funding constraints [50, 55]. These studies included qualitative studies conducted to explore the advantages and challenges of the program. One of the indicated advantages of the SFP program is the contribution of the program in saving parents money and time as the SFPs were making use of (purchasing) local food and agricultural development. Despite the advantage of SFP, studies highlighted challenges related to food provision, infrastructure, and administration.

Challenges related to food provision included a lack of hygienic, adequate, regular, and quality food. Infrastructure challenges, including a lack of independent SFP structures at various levels making implementation and sustainability challenging; a lack of training for cooks; a lack of physical capital, such as feeding utensils, electricity, and water, exacerbated by administration problems, such as inadequate stakeholder engagement, absence of clear policy and financial constraints were reported. Table 4.

**Table 4:** Summary of studies on other variables of school feeding programs in Ethiopia

No	Authors	Year	Aims/objective of the study	Study design	Key findings on other variables
1	Desalegn TA, Gebremedhin S, and Stoecker BJ. [45]	2022	To explore the successes and challenges of the SFP	Exploratory qualitative study	Local purchase of food or agricultural development (home-grown SFP) contribution in saving the parents’ money and time Lack of permanent clean water provision, delay in ration delivery, poor-quality food provision, inadequate amount of food allocated for the academic year, lack of necessary infrastructure for the program, and lack of training in sanitation and hygiene for cooks were among the major challenges identified in SFP
2	Sertse A. [55]	2019	To assess the overall situation of school feeding program in Government Elementary Schools	Qualitative study / focus group discussion	Obstacles of SFP • a lack of awareness • financial limitations, the fact that the amount of money allocated to feed one child does not take into account the current state of the market, • physical capital (the lack of feeding utensils, the feeding hall's lack of electricity, the lack of water • policy-related difficulties (no clear policy about administration of tax and payments for the poor women cooks • the participation of stakeholders is insignificant compared with the need of needy students) Even though the food varies from Monday to Friday, it does not include foods like milk, fruit, and other nourishing items
3	Haile Y and Ali A. [50]	2019	To explore the sustainability of school feeding program	Descriptive quantitative and qualitative	Difficulties, for implementation and sustainability • an increase in the number of students from year to year, a lack of community and government support, • lack of sectorial involvement, • lack of institutional and financial capacity • lack of independent SFP structures at various levels
4	Lakew B. [39]	2021	To investigate the adequacy of meal macronutrients, dietary diversity, body composition, and the iron status of school children aged between 7–14	A cross-sectional study	Food variety and dietary diversity scores among schoolchildren fell short of the Food and Agriculture Organization's suggested levels

## Discussion

This scoping review aims to map the evidence relating to school feeding programs and their potential role in managing children under nutrition. The main objectives are to explore school feeding programs coverage, quality, nutritional impacts, funding issues, and its impact on educational achievements among students in Ethiopia. The finding of the study indicates mixed results.

School feeding shows an improvement in BMI among underweight students. Additionally, body fat is increased among thin students. Furthermore, the hemoglobin status increased among anemic students. There are also increased weight and height [30–35]. This shows that school feeding programs improve the overall anthropometric status. A study from South Africa also obtainedsimilar results. The school breakfast programme improved anthropometric measurements with a 10% increase in the number of children within the healthy BMI range for their age [56] Similarly, a study in Kenya reported that the school feeding programme clearly had a positive effect on children’s nutritional status. The programme reduced anemia and malnutrition and improved child growth [57] However, some studies have shown that SFP has no effect on stunting, thinness, weight, and hemoglobin level [33, 35, 37, 38]. Similarly, a study reported that caloric and nutritional contributions were less than two-thirds of the daily RNIs needed from school meals [36]. This signifies that there is a need for further study.

School feeding programs improve academic performance, [34, 40, 41, 45] increase class attendance, [30, 31, 40, 43–47, 49, 50] decrease the dropout rate, [42–44, 49] and increase enrollment. [40, 42, 48, 50] The majority of studies have reported favorable results on the importance of school feeding programs for educational outcomes [30, 31, 34, 40–45, 47, 48, 50]. Different studies in different countries also show consistent results with this study. A study in Uganda revealed that, SFP had large impacts on school attendance, and reduced grade repetition [58]. Nutritional interventions in school-aged children have been reported to result in improved cognitive function [9–11]. Furthermore, this study found that meals provided in the morning help students better than those provided at the end of school [41]. A similar study in South Africa revealed that meals served at breakfast are also shown to have a positive impact [56] Children who consume a meal before learning have better short-term memory function, as the brain activated differently based on nutrient supply [59] However, some studies indicate that, SFP has no effect on attendance, dropout rate and academic achievement [34, 46, 48, 51–54]. If loss of teaching time could be the factor for no significant difference in achievement test scores, it might be simpler to offer a take home ration program to prevent disturbance during the school day [60]. This indicates the need for additional research.

There were also advantages and challenges of the school feeding program. Advantages of the SFP programs are the contribution of the program in saving parents money and time, as the SFPs were making use of (purchasing) local food and agricultural development. Nevertheless, the challenges related to food provision included a lack of hygienic, adequate, regular, and quality food. Infrastructure challenges, including a lack of independent SFP structures at various levels making implementation and sustainability challenging; a lack of training for cooks; and a lack of physical capital, such as feeding utensils, electricity, and water exacerbated by administration problems, such as inadequate stakeholder engagement; and the absence of clear policy and financial constraints [50, 55].

There is a shortage of evidence on SFPs in Ethiopia. The majority of the studies identified were cross-sectional studies. Therefore, there is a need to conduct more research using higher quality study designs and quantitative research for decision and policy-making.

The limitations of this study were low quality of study design, primarily, cross sectional. Although the study results are mainly from low study designs, the findings of this review have the first hand information to inform the importance of school feeding programs and their effect on nutritional status and academic performance.

## Conclusion

School feeding programs in Ethiopia showed mixed findings on nutritional status and academic performance. Besides, poor-quality food provisions and financial or funding constraints affect school feeding programs. The SFP should take into account the nation's diversity, including its geography, climate, agrarian and pastoral areas. Although there are studies conducted in different areas of the country and schools, it is important to conduct nationwide study to conclusively determine the coverage, nutritional, and educational effect of the SFP in Ethiopia.

### Strengths and limitations of this study

This is the first scoping review on school feeding in Ethiopia. The search strategy included four electronic databases, including ancestor searching and grey literature (both government and organization websites). Additional literature was sought from relevant bodies such as the ministry of education and experts on the area. This scoping review provides information on the range and nature of evidence on school feeding in Ethiopia and identifies research gaps. Nevertheless, the study was limited to reviewing findings only from publications and grey literature with scientific methods and might therefore miss important information from other sources.

## Data Availability

The datasets used and/or analyzed during the current study are available from the corresponding author upon reasonable request.

## Abbreviations

ASLI: Africa Student Learning Index
BMI: Body Mass Index
ERIC: Education Resources Information Centre
FAO: Food and Agriculture Organization
3ie: International Initiative for Impact evaluation
SFP: School Feeding Program
UNESCO: United Nations Educational, Scientific and Cultural Organization
UNICEF: United Nations International Children's Emergency Fund
WFP: World Food Programme
WHO: World Health Organization
